# Protective Effect of Mycosporine-like Amino Acids Isolated from an Antarctic Diatom on UVB-Induced Skin Damage

**DOI:** 10.3390/ijms242015055

**Published:** 2023-10-10

**Authors:** Kai Wang, Yashan Deng, Yingying He, Junhan Cao, Liping Zhang, Ling Qin, Changfeng Qu, Hongmei Li, Jinlai Miao

**Affiliations:** 1 Key Laboratory of Marine Eco-Environmental Science and Technology, First Institute of Oceanography, Ministry of Natural Resources, Qingdao 266061, China; wk2303@stu.ouc.edu.cn (K.W.); threethree714@outlook.com (Y.D.); heyinging@fio.org.cn (Y.H.); caojunhan@stu.ouc.edu.cn (J.C.); 15689487176@163.com (L.Z.); qinling@fio.org.cn (L.Q.); cfqu@fio.org.cn (C.Q.); 2 Laboratory for Marine Drugs and Bioproducts, Qingdao Pilot National Laboratory for Marine Science and Technology, Qingdao 266237, China; 3 Marine Natural Products R&D Laboratory, Qingdao Key Laboratory, Qingdao 266061, China; 4 Key Laboratory of Biomedical Polymers, Shandong Academy of Pharmaceutical Science, Jinan 250100, China; yijianmei2003@163.com

**Keywords:** mycosporine-like amino acids, Antarctic diatom, ultraviolet B, skin damage, oxidative stress, inflammation

## Abstract

Although it is well recognized that mycosporine-like amino acids (MAAs) are ultraviolet (UV) protective agents that can reduce UV damage, the specific biological mechanism of its role in the skin remains unclear. In this study, we investigated the effect of MAAs extracted from Antarctic diatom *Phaeodactylum tricornutum* ICE-H on UVB-induced skin damage using a mice model. The MAAs components identified by liquid chromatography-tandem mass spectrometry included 4-deoxygadusol, shinorine, and porphyra-334, which were purified using a Supledean Carboxen1000 solid phase extraction column. The antioxidant activities of these MAA compounds were tested in vitro. For UVB-induced skin photodamage in mice, MAAs alleviated skin swelling and epidermal thickening in this study. We detected the content of reactive oxygen species (ROS), malondialdehyde, and collagen in skin tissue. In addition, quantitative real-time polymerase chain reaction was used to detect nuclear factor-κB (NF-κB), tumor necrosis factor α, interleukin-1β, cyclooxygenase-2, mitogen activated protein kinase (MAPK) family (extracellular signal-regulated kinase, c-Jun amino-terminal kinase, and p38 kinase), and matrix metalloproteinases. The expression of these cytokines and enzymes is related to inflammatory responses and collagen degradation. In comparison to the model group without MAA treatment, the MAA component decreased the concentration of ROS, the degree of oxidative stress in the skin tissue, and the expression of genes involved in the NF-κB and MAPK pathways. In summary, these MAA components extracted from *Phaeodactylum tricornutum* ICE-H protected against UVB-induced skin damage by inhibiting ROS generation, relieving skin inflammation, and slowing down collagen degradation, suggesting that these MAA components are effective cosmetic candidate molecules for the protection and therapy of UVB damage.

## 1. Introduction

The majority of individuals are inevitably exposed to sunlight each day, and the skin, the body’s first line of defense, is the most crucial organ for the absorption ultraviolet (UV) radiation. UV is useful in that it helps the body synthesize vitamin D, but it can also cause skin photodamage. The solar ultraviolet radiation (UVR) reaching the Earth’s surface includes UVA (320–400 nm) and UVB (280–320 nm) [1]. UVR of various wavelengths has distinct effects on the skin. UVB is mostly absorbed by the skin’s epidermis and causes acute photodamage such as erythema and inflammation, which can eventually lead to photoaging and skin cancer. To prevent photodamage, additives have been used or recommended to enhance UV protection in sunscreens. However, the ingredients of sunscreens must be consistent with human safety and environmental safety, meaning that the development of the ideal sunscreen still needs further innovation and research.

UVB stimulates a significant increase in intracellular reactive oxygen species (ROS) generation, activating a variety of signal pathways involving oxidative stress, skin inflammatory, and DNA damage [2]. ROS not only cause cell death and lipid peroxidation, but also activate nuclear factor-κB (NF-κB) and activator protein-1 via mitogen activated protein kinase (MAPK) signaling pathways, and up-regulates the expression of matrix metalloproteinases (MMPs), resulting in skin extracellular matrix (ECM) degradation and photoaging skin relaxation and wrinkles. NF-κB induces the expression of proinflammatory cytokines such as tumor necrosis factor (TNF)-α and interleukin 1 (IL-1) [3], which further aggravate the accumulation of ROS and the degradation of ECM. ROS also lead to the overexpression of the inflammatory cascade reaction, which enlarges related enzymes such as cyclooxygenase-2 (COX-2) [4]. As a result, limiting ROS generation and accumulation is critical for preventing or treating skin damage.

Many marine and freshwater organisms including fungi [5], cyanobacteria [6], macroalgae [7,8], microalgae [9], and aquatic animals (e.g., corals [10], sponges [11], sea urchins [12] and fishes [13]) have accumulated compounds with UVR absorption capacity, mycosporine-like amino acids (MAAs), which enable them to protect themselves due to long-term exposure to high levels of UVR. MAA is a nitrogen-containing secondary metabolite, which is soluble in water. MAAs typically have a maximum absorption wavelength of between 268 and 362 nm and a high molar extinction coefficient of between 28,100 and 50,000 M^−1^ cm^−1^. Since MAAs have good stability and antioxidant, anti-inflammatory, and photoprotective activities that enable organisms to resist UV radiation, they are regarded as the best choice for sunscreen compounds [14]. Cyclohexene ketone rings or cyclohexene imine rings coupled with amino acids, amino alcohols, or amino groups make up the majority of MAAs. So far, over 40 different types of MAA have been found. Among these, porphyra-334, shinorine, and mycosporine-glycine have attracted the most attention in photochemistry [15]. After absorbing UV, these molecules release energy in the form of heat without producing ROS [16]. Although there is evidence that MAAs are the main candidates for photoprotective molecules used by humans, little research has been conducted using skin as a model to investigate their photoprotective potential and mechanisms [17,18,19].

The harsh natural environment and climatic characteristics of Antarctic sea ice, such as long-term low temperature, ice and snow cover, and significant UVR, provide numerous obstacles to the life limit [20]. Antarctic ice microalgae is a broad term for unicellular microalgae found in the Antarctic region. These microalgae are the principal body and source of primary productivity in the Antarctic sea ice ecosystem, which is frequently dominated by diatoms [21]. Ice algae have developed special resistance properties to adapt to the polar sea ice environment with high osmotic pressure and intense UV exposure. It was discovered that the accumulation of MAAs in diatoms increased as the intensity of UV radiation rose [22,23]. There is currently a scarcity of research on the biological activity of MAAs in Antarctic diatoms. In this study, MAAs were extracted from the Antarctic diatom *Phaeodactylum tricornutum* ICE-H and applied to a mice skin model to explore their protective mechanism against skin photodamage

## 2. Results

### 2.1. The Characterization of MAAs Extracted from Phaeodactylum tricornutum ICE-H

**Figure 1:**
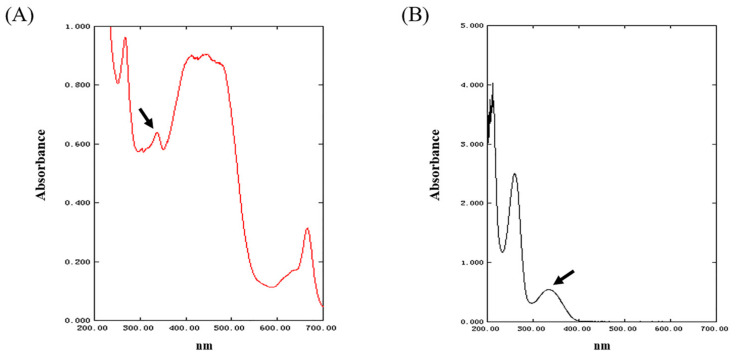
Ultraviolet–visible absorption spectra of MAAs in *Phaeodactylum tricornutum* ICE-H: (**A**) absorption spectra of MAAs before purification at 200–700 nm; (**B**) absorption spectra of MAAs after purification at 200–700 nm. The arrow refers to the absorption peak of the extract at 300–400 nm.

The UV spectrum of the MAAs in *Phaeodactylum tricornutum* ICE-H is shown in Figure 1. The spectrum showed that the sample had a clear absorption peak between 300 nm and 400 nm, and it was tentatively determined that MAAs extracted from this diatom might contain some disubstituted MAAs with UV absorption (such as shinorine, porphyra-334, and asterina-330) [24,25]. As shown in Figure 1A, after the initial removal of pigments by chloroform, the crude extract still contained a large quantity of carotenoids and luteolin with absorption around 400–500 nm, as well as chlorophyll and phycocyanin with absorption peaks at 600–700 nm. The spectral scan of the eluate in Figure 1B shows that the absorption peaks at 400–500 nm and 600–700 nm disappeared, and the whole spectrum had fewer spurious peaks, indicating that the pigments and other impurities could be effectively removed using the solid-phase extraction method to obtain a relatively pure MAA sample.

**Figure 2:**
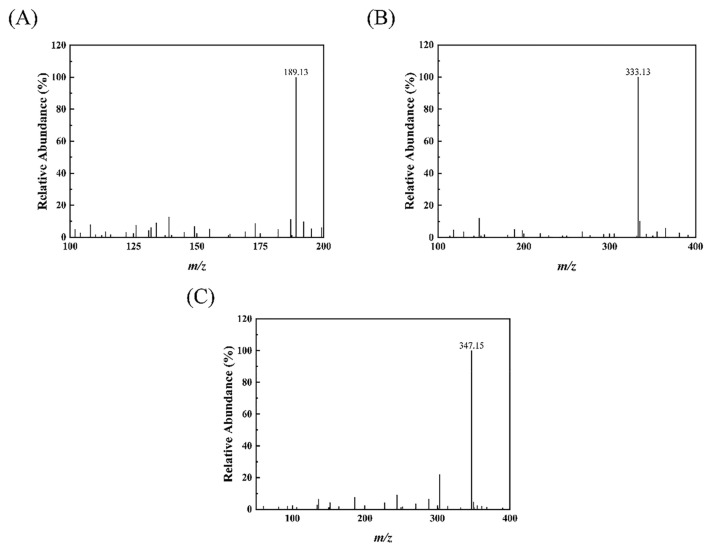
MS spectra of MAA compounds: (**A**) 4-deoxygadusol; (**B**) shinorine; (**C**) porphyra-334.

**Figure 3:**
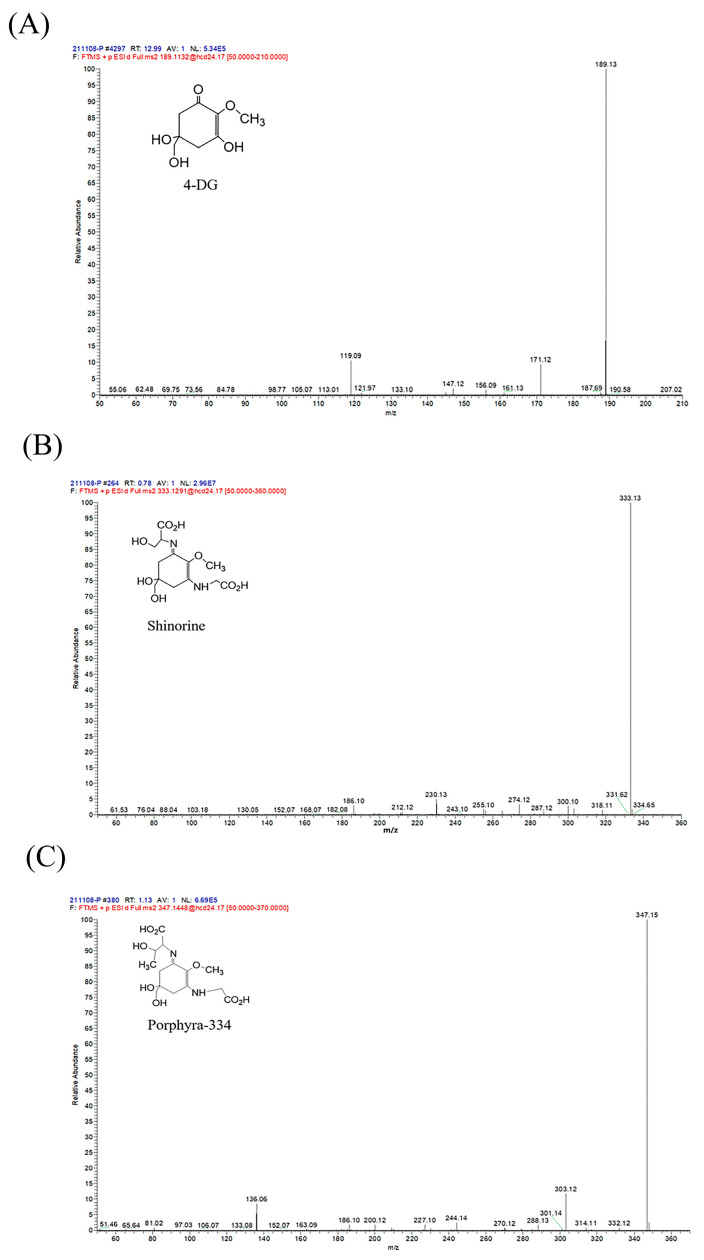
Fragmentation patterns of MAAs by MS/MS in extracts of *Phaeodactylum tricornutum* ICE-H. The mass spectra indicate the main fragmentation pattern in 4-deoxygadusol (4-DG) (**A**), shinorine (**B**), and porphyra-334 (**C**).

Mass spectral analysis of MAA compounds identified molecular ions *m*/*z* 189.13, 333.13, and 347.15 [M + H]^+^, respectively (Figure 2). The compounds were further analyzed by liquid chromatography-tandem mass spectrometry. According to the results of secondary fragments (Figure 3), the ions of the compound in Figure 3B included *m*/*z* 300.10, *m*/*z* 274.12, *m*/*z* 230.13, and *m*/*z* 186.10. The ions of the compound in Figure 3C included *m*/*z* 303.12, *m*/*z* 288.13, *m*/*z* 244.14, *m*/*z* 200.12, *m*/*z* 186.10, and *m/z* 136.06. Manyun Chen et al. [26] analyzed the MAA components in the heterologous expression products of *Escherichia coli*, and the fragments of shinorine and porphyra-334 were basically consistent with the main fragments in this experiment. By comparing mass spectra from various reports, it is discovered that *m*/*z* 288, *m*/*z* 244, *m*/*z* 230, *m*/*z* 197, *m*/*z* 186, and *m*/*z* 137 are common molecular ion peaks after MAA compound cleavage [26,27]. After comparison, the MAA components in *Phaeodactylum tricornutum* ICE-H were identified to be 4-deoxygadusol (4-DG) (Figure 3A), shinorine (Figure 3B), and porphyra-334 (Figure 3C).

### 2.2. Antioxidant Levels of MAAs

**Figure 4:**
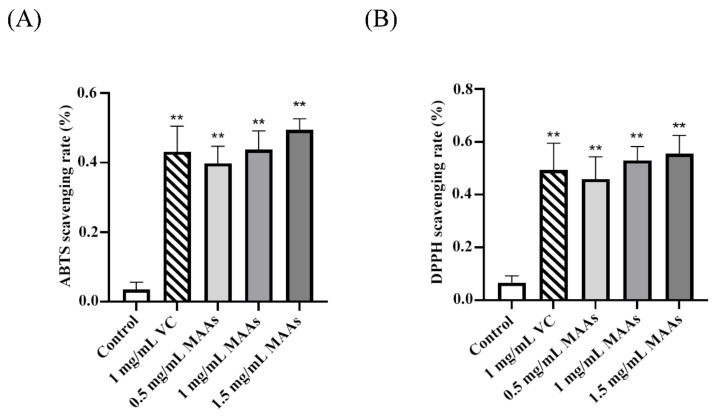
Free radical scavenging test of MAAs in vitro: (**A**) determination of ABTS scavenging rate; (**B**) determination of DPPH scavenging rate. DPPH, di (phenyl)-(2,4,6-trinitrophenyl) imino azanium; ABTS, 2,2′-azino-bis (3-ethylbenzothiazoline-6-sulphonic acid); control, ultrapure water; VC, vitamin C. The data are denoted as the mean ± SD from triplicate results (** *p* < 0.01, compared with the control).

As shown in Figure 4, MAAs could considerably scavenge ABTS and DPPH free radicals when compared to the control group (*p* < 0.01), and the free radical scavenging ability of MAAs increased with concentration.

### 2.3. MAAs Ameliorated Photodamage in Mice Skin

**Figure 5:**
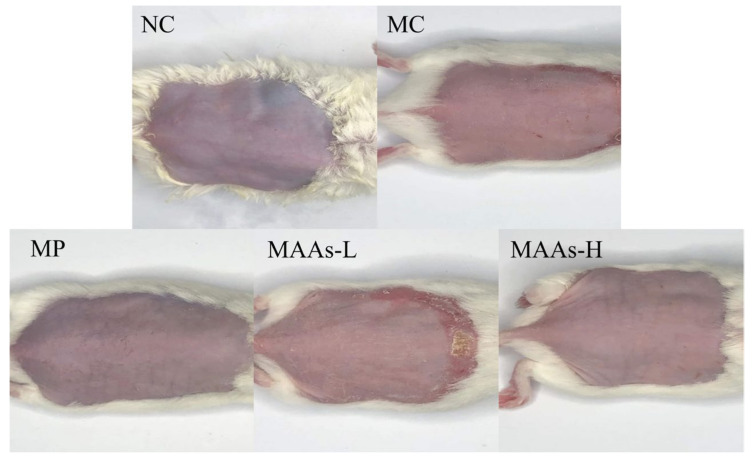
The skin’s visible differences in UVB-induced mice skin damage. NC represents the normal group, MC represents the model group, MP represents the positive group, MAAs-L represents the low-dose MAA group, MAAs-H represents the high-dose MAA group.

As shown in Figure 5, UVB radiation caused skin erythema. Compared with the model group (MC), the erythema and UV damage in the positive group (MP, vitamin C) were significantly reduced, and the damage in the low-dose MAA group (MAAs-L) was slightly improved. The erythema in the high-dose MAA group (MAAs-H) was greatly reduced, as were UV damage and keratosis.

**Figure 6:**
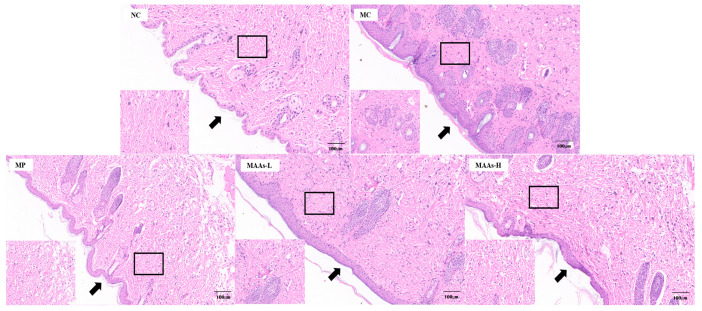
Hematoxylin−eosin staining of mouse dorsal skin following UVB irradiation (50× magnification). Inside the box is the reticular layer of the dermis, and arrows indicate epidermal thickness. NC represents the normal group, MC represents the model group, MP represents the positive group (vitamin C), MAAs-L represents the low-dose MAA group, MAAs-H represents the high-dose MAA group.

We performed histopathological analysis of skin lesions by hematoxylin−eosin (H&E) staining. The results are shown in Figure 6. The back skin of mice in the NC group without UVB irradiation showed a complete and clear structure. However, the mice in the MC group irradiated by UVB showed obvious thickening of the epidermis. Compared with the NC group, the skin of the MP group and MAA group also showed epidermal thickening, but the degree of thickening was significantly smaller.

### 2.4. MAAs Reduced Oxidative Stress Levels in Mice Skin

**Figure 7:**
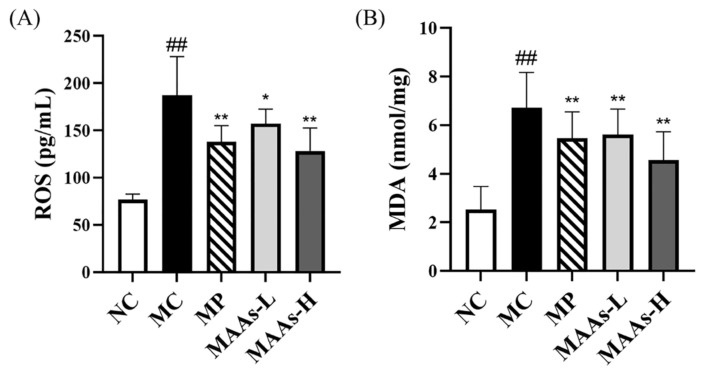
Effects of MAAs on ROS generation (**A**) and MDA levels (**B**) in UVB-induced mice skin. ROS, reactive oxygen species; MDA, malondialdehyde. NC represents the normal group, MC represents the model group, MP represents the positive group, MAAs-L represents the low-dose MAA group, MAAs-H represents the high-dose MAA group. The data are denoted as the mean ± SD from triplicate results. (^##^ *p* < 0.01, compared with the NC group; * *p* < 0.05, ** *p* < 0.01, compared with the model group).

The generation of ROS and its metabolite malondialdehyde (MDA) were detected, and the results are shown in Figure 7. After UVB irradiation, ROS generation in the skin of mice in the MC group was considerably higher than that in the NC group (*p* < 0. 01). After the intervention of vitamin C (VC) and MAAs, the content of ROS produced in the skin of mice was significantly reduced, and was lower in the MAAs-H group than in the MAAs-L group. Moreover, UVB radiation damage substantially raised MDA content in the skin of the MC group compared to the NC group (*p* < 0.01). However, MAA treatment caused a significant decrease in MDA content in the skin of mice (*p* < 0.01).

### 2.5. MAAs Inhibited Skin Inflammation in UVB-Irradiated Mice

**Figure 8:**
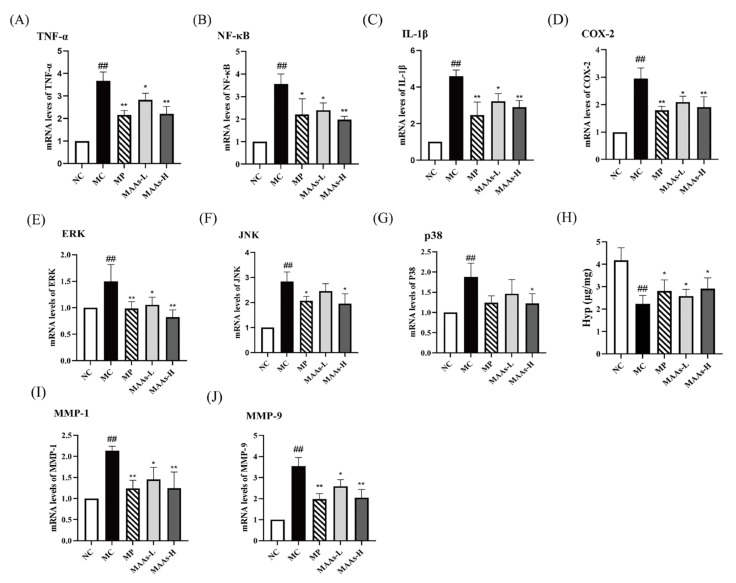
The effects of MAAs on the mRNA levels of inflammation and skin degradation-related genes and the content of hydroxyproline (Hyp) in skin tissue: (**A**–**D**) mRNA expression of TNF-α, NF-κB, IL-1β, and COX-2; (**E**–**G**) mRNA expression of ERK, JNK, and p38 kinase; (**H**) the content of Hyp; (**I**,**J**) mRNA expression of MMP-1 and MMP-9 (^##^ *p* < 0.01, compared with the NC group; * *p* < 0.05, ** *p* < 0.01, compared with the model group). NC represents the normal group, MC represents the model group, MP represents the positive group, MAAs-L represents the low-dose MAA group and MAAs-H represents the high-dose MAA group. NF-κB, nuclear factor-κB; COX-2, cyclooxygenase-2; TNF-α, tumor necrosis factor-α; IL-1, interleukin 1; MMP-1, matrix metalloproteinases-1; ERK, signal-regulated kinase; JNK, c-Jun amino-terminal kinase.

To further verify the protective effect of MAAs against skin inflammation, we measured the levels of several inflammatory cytokines in skin tissues. According to the results of real-time polymerase chain reaction (qRT-PCR) (Figure 8A–D), the mRNA expressions of IL-1β, TNF-α, NF-κB, and COX-2 all showed marked increases in the skin of MC mice compared to that of the NC mice. Interestingly, the applications of VC and MAAs both significantly inhibited the expression of IL-1β, TNF-α, NF-κB, and COX-2 (*p* < 0.05, *p* < 0.01).

### 2.6. MAAs Reduced Skin Collagen Degradation in UVB-Irradiated Mice

As shown in Figure 8E–G, compared with the NC group, the mRNA expression of signal-regulated kinase (ERK), c-Jun amino-terminal kinase (JNK), and p38 kinase in the MAPK family were significantly up-regulated after UVB damage in the skin of mice in the MC group (*p* < 0.01), while the skin treatment with VC and MAAs obviously reduced ERK expression (*p* < 0.05 or *p* < 0.01), treatment with VC and high-dose MAAs significantly reduced JNK expression (*p* < 0.05), and treatment with high-dose MAAs significantly reduced p38 expression (*p* < 0.05). According to the results of qRT-PCR (Figure 8I,J), UVB radiation significantly increased the expression of MMPs (MMP-1 and MMP-9) in the skin of mice (*p* < 0.01). However, after treatment with VC and MAAs, there was a significant decrease in the expression levels of MMPs in UVB-irradiated skin (*p* < 0.05 or *p* < 0.01). As shown in Figure 8H, compared with the NC group, the hydroxyproline (Hyp) of skin content in the MC group was significantly reduced (*p* < 0.01), while mice in the MP, MAAs-L, and MAAs-H groups showed a significant increase in Hyp content after treatment with VC and MAAs (*p* < 0.05).

## 3. Discussion

Sunscreen is a popular approach for individuals to protect themselves against ultraviolet radiation. However, the safety and environmental protection of sunscreen chemicals is currently a major challenge. Some sunscreen compounds have been linked to neurotoxicity [28], endocrine disruption, and other human health issues, while others are difficult to degrade, causing coral bleaching and other negative environmental effects [29,30]. Therefore, the development of green, non-toxic, and biocompatible sunscreen chemicals offers enormous research and innovation potential. Among marine natural products, MAAs have emerged as the most promising new generation of sunscreens. In addition to photoprotection, MAAs exhibited a variety of biological actions including antioxidant [31,32], anti-inflammatory [33], and tumor cell growth inhibition [34] properties. Furthermore, MAAs are non-toxic and light stable. However, there are currently few studies on the protective mechanism of MAAs on skin photodamage, and in vitro studies [33,35,36] comprise the majority of these. This study revealed that the extracts of MAAs from the Antarctic diatom *Phaeodactylum tricornutum* ICE-H can reduce the skin damage caused by UVB in mice and thoroughly investigated the protective mechanism of MAAs on skin photodamage, which is advantageous in the prevention of skin photoaging and skin cancer.

The separation of MAAs in marine algae has been reported since the 1990s [37]. In addition to macroalgae, marine microalgae are also a source of MAA production due to their rapid reproduction and ease of cultivation. About 152 species of marine microalgae have been found to produce MAAs, and studies on MAAs in cyanobacteria were conducted earliest and most often, followed by those in dinoflagellates [9]. However, there are no studies on the structure and activity of MAAs in Antarctic diatoms, especially *Phaeodactylum tricornutum*. In this study, we isolated and examined the composition of MAAs in the Antarctic diatom *Phaeodactylum tricornutum* ICE-H. We revealed that this MAA’s compounds reduced skin damage caused by UVB in mice, and thoroughly investigated the protective mechanism of MAAs on skin photodamage, which is advantageous in the prevention of skin photoaging and skin cancer. There are three previous reports on the photoprotective effect of MAAs on mice skin. These MAA compounds were isolated from red algae, namely *Porphyra yezoensis*, *Porphyra tenera*, and *Porphyra rosengurttii*, respectively. F. de la Coba et al. investigated the effect of MAAs on the expression of heat shock protein-70 in 2009 [19]. Hou et al. proved that MAAs reduced the expression of MMP-1, MMP-3, and TNF-α, and activated the NF-κB pathway in photoaging skin to participate in the synthesis of matrix collagen [17]. In 2019, Hou et al. proved that MAAs inhibited the reduction of endogenous antioxidant enzymes, thereby inhibiting UV-induced skin photoaging [18]. In addition to the NF-κB pathway and the expression of MMPs mentioned above, MAA compounds in this study showed significant free radical scavenging ability in vitro, ROS scavenging ability in skin, and regulation of the MAPK pathway. In this paper, the protective mechanism of MAAs against skin photodamage was studied more comprehensively.

UVB radiation can lead to the thickening of folds and red spots on the surface of skin, which is one of the most visible indications of photodamage [19,38]. In this study, MAAs from *Phaeodactylum tricornutum* ICE-H significantly improved the redness and keratinization of the back skin of mice induced by UVB, as previously reported for MAAs (porphyra-334 and shinorine) extracted from red algae on the skin repair of hairless mice [19]. In addition to the visible improvement of skin condition, the results of H&E-stained tissue sections revealed that MAAs attenuated UVB-induced increase in epidermal thickness. These results showed that MAAs extracted from Antarctic ice algae *Phaeodactylum tricornutum* had a protective effect against skin damage caused by UVB radiation.

The primary effect of UV on skin is the generation of ROS, which induce oxidative damage to protein, lipid, and nucleic acid in cells and operate as a second messenger to affect many signaling pathways in the cell [39]. The primary focus for skin protection lies in the efficient clearance of excessive ROS, thus emphasizing the crucial role of antioxidant defense mechanisms. We evaluated the antioxidant capacity of MAAs in vivo and in vitro. The ABTS and DPPH free radical scavenging techniques, which follow the electron transfer reaction mechanism [40], are commonly used to assess the antioxidant activity of active compounds in vitro [41]. MAAs isolated from *Phaeodactylum tricornutum* ICE-H showed considerable free radical (ABTS and DPPH) scavenging ability in this investigation, showing that they have strong antioxidant capability in vitro. MDA, a major ROS metabolite in the body, is the end product of lipid peroxidation in vivo, and its synthesis aggravates membrane damage [42]. MDA content is an essential indicator of the body’s potential antioxidant capacity as it can represent the rate and intensity of lipid peroxidation in the body, as well as the extent of tissue peroxidation damage [17]. According to this study, UVB radiation significantly increased the amount of MDA in mice skin. However, after receiving MAAs, the amount of MDA in the mice skin was significantly reduced, indicating that both the rate and intensity of lipid peroxidation in the mice back skin were reduced, as well as the degree of skin photodamage. Furthermore, excessive ROS in skin can activate the NF-κB pathway, consequently activating the transcription factor NF-κB and affecting the production and release of various pro-inflammatory cytokines such as TNF-α, IL-2, IL-6, and IL-1β [43,44]. Many studies have discovered that UVB radiation increases the expression of pro-inflammatory gene COX-2 in skin surface cells, which may also play a role in the development of skin cancer [45,46]. In this study, MAA treatment suppressed the expression of transcription factor NF-κB and the gene COX-2, as well as the production of inflammatory cytokines TNF-α and IL-1β, indicating that MAAs alleviated the skin inflammatory damage caused by UVB.

In addition to the NF-κB pathway, ROS also activate MAPK cascades, including ERK, JNK, and p38 kinase, promote the expression of MMPs, lead to the rapid degradation of collagen, and ultimately accelerate skin aging and even skin cancer [47]. In this study, MAAs modulated MAPK signaling, inhibited the up-regulation of MMP-1 and MMP-9, and had a significant effect on the loss of collagen caused by UVB. Collagen is the most essential component of animal connective tissue, accounting for around one-third of the body’s protein. The degradation of collagen in the skin is one of the reasons for wrinkle formation in skin photoaging [48]. MMP-1 is a collagenase that causes collagen degradation in UV-damaged skin [1], while MMP-9 is a gelatinase that degrades denatured collagen [49]. As a representative amino acid of skin collagen, the content of Hyp can reflect the change in collagen in skin [50]. In this study, we measured the amount of Hyp in the skin to determine the degree of collagen loss. The result indicated that MAAs had a significant effect on the loss of collagen caused by UVB.

Most commercially available sunscreens now have a minimal effect on lowering visible-light-induced ROS, and so cannot protect the skin from visible-light-induced responses [51]. Shinorine and porphyra-334 have been shown to be capable of absorbing UV and dispersing the absorbed energy as heat without generating ROS [52,53]. Second, MAAs, a type of antioxidant, can also prevent or eliminate ROS from cells. In this study, we measured the content of ROS, and found that UVB radiation caused excessive ROS generation in the skin of mice, while the ROS content was significantly reduced by MAA treatment. However, the low content of MAAs directly extracted from diatoms presents a challenge for large-scale preparation, prompting us to investigate the biosynthesis genes of MAAs in *Phaeodactylum tricornutum* and pursue high-purity yields through heterologous expression in future studies.

## 4. Materials and Methods

### 4.1. Materials and Reagents

The Antarctic diatom (*Phaeodactylum tricornutum* ICE-H) used in this experiment was isolated and purified from seawater and sea ice near the Zhongshan Research Station of Antarctica (S 69°48′, E 77°48′) during China’s 18th Antarctic Scientific Expedition [54]. *Phaeodactylum tricornutum* ICE-H was cultivated in a low-temperature light incubator after being infected in a triangle flask with f/2 media [55] at a 30% inoculation level. The light cycle was 12 L:12 D, the light intensity was 40 μmol m^−2^ s^−1^, and the culture temperature was 4 ± 1 °C. The reagent kits for the measurement of ROS, MDA, Hyp, ABTS, and DPPH were purchased from Servicebio Technology Co., Ltd. (Wuhan, China). SPF male Kunming (KM) mice were purchased from Pengyue Experimental Animal Breeding Co., Ltd. (Jinan, China). The primers targeting mRNAs, including IL-1β, TNF-α, NF-κB, COX-2, MMP-1, MMP-9, and β-actin, were synthesized by Sangon Biotech Co., Ltd. (Shanghai, China).

### 4.2. Preparation of MAAs

*Phaeodactylum tricornutum* ICE-H cells were extracted by centrifugation at 6000 rpm for 10 min at 4 °C and then freeze-dried to obtain algal powder. The powder was extracted in methanol (Sinopharm Chemical Reagent Co., Ltd., Beijing, China, 10 mL methanol per 1 g) at 4 °C overnight. Afterward, the sample was centrifuged (8000 rpm, 20 min) at 4 °C, and the supernatant was collected, evaporated to dryness at 30 °C, and redissolved with ultrapure water. Chloroform (Sinopharm Chemical Reagent Co., Ltd., Beijing, China) was added in the supernatant with a ratio of 3:2 (water:chloroform) and mixed. After being centrifuged (8000 rpm), the upper solution was collected and scanned in an ultraviolet spectrophotometer (Beijing Persee General Instrument Co., Ltd., Beijing, China) at a wavelength of 200 to 400 nm.

The MAA crude sample was purified using a Supledean Carboxen1000 solid phase (Thermo Fisher Scientific Inc., Shanghai, China) extraction column. The samples filtered using a 0.45 μm membrane were injected into the column at a flow rate of 1 mL/min, and the effluent was collected. The column was washed with 1 mL of ultrapure water, and the water in the column was drained after the liquid was dried. Finally, 6 mL of 80% acetonitrile was injected twice, and the elution was completed. The eluent was collected, evaporated to dryness, redissolved with ultrapure water, and lyophilized at −80 °C. The freeze-dried sample was redissolved with ultrapure water, and then scanned again with an ultraviolet spectrophotometer (Ultrospec 2100, BIOCHROM, USA) at a wavelength of 200 to 400 nm.

### 4.3. Identification and Characterization of MAAs

The purified sample (5 mg) was dissolved in 1 mL of methanol, water, and formic acid in the ratio of 15:4:1 (*v*:*v*:*v*). After centrifugal purification and enrichment, it was dried by nitrogen blowing, redissolved with 80% acetonitrile-water solution (100 μL), and filtered through PTFE membrane (0.22 μm) for ultra performance liquid chromatography-tandem mass spectrometry (UPLC-MS/MS). The UPLC-MS/MS system contains Thermo Vanquish UHPLC Thermo Fisher Scientific and Q-Exactive HF Thermo Fisher Scientific. The specific experimental conditions are as follows: chromatographic column: Zorbax Eclipse C18 (1.8 μm × 2.1 mm × 100 mm); mobile phase: ammonium acetate aqueous solution (5 mmol/L) containing 0.1% formic acid (A) and methanol (B), with a flow rate of 0.3 mL/min. The linear gradient conditions were as follows: starting with 5% solvent B, then ramped linearly to 80% at 15 min. The column temperature was 35 °C and the injection volume was 2 μL.

### 4.4. Detection of Antioxidant Level of MAAs In Vitro

Scavenging activity of MAAs (0.5 mg/mL, 1 mg/mL, and 1.5 mg/mL) on DPPH and ABTS was assessed according to the instructions of the test kits (Solarbio, BC4755 and BC4775). For DPPH measurement, MAAs and vitamin C were mixed with ethanol containing 25% DPPH and reacted at room temperature for 30 min in the dark. Finally, the absorbance was read at 515 nm. For ABTS measurement, MAAs and vitamin C were mixed with ABTS liquid and allowed to stand at room temperature in the dark for 6 min. Finally, the absorbance was read at 405 nm.

### 4.5. Establishment of UVB-Induced Skin Photodamage Model

All animal experiments met the requirements of the National Laboratory Animal Ethics Committee of China and were approved by the Animal Care Review Committee (approval number SYXK2020-0422), Qingdao University of Science and Technology, China. After 7 days of adaptive feeding, 30 mice (6–7 weeks) were evenly divided into 5 groups based on body weight, with 6 mice in each: normal group (NC), model group (MC), positive group (MP), MAAs of low- and high-dose groups (MAAs-L, MAAs-H). The back of the mice was depilated with ethanol solution containing Na_2_S (6%, *w*/*w*), and the depilation area was 2 × 2 cm^2^, followed by adaptive feeding for 3 days. Then, the mice in the MC group were treated with 200 μL distilled water on the back; the MP group was treated with 200 μL VC (1 mg/mL); the MAAs-L group was treated with 200 μL MAAs (2.5 mg/mL); and the MAAs-H group was treated with 200 μL MAAs (5 mg/mL). Dose determination was based on the previous study [44]. These treatments were repeated twice a day. According to the previous research, the UVB (302–310 nm) intensity was 300 mJ/cm^2^ [56]. The NC group received no UV irradiation and only 200 μL of distilled water for 7 days. The body weight of mice was recorded every day, and the skin condition of the mice back was observed. After the last irradiation, the mice were sacrificed after the back state was photographed, and the back skin was collected and preserved at −80 °C or fixed with 4% paraformaldehyde (Sinopharm Chemical Reagent Co., Ltd., Beijing, China) for further analysis.

### 4.6. Histological Analysis of Skin Tissues

The fixed tissues were placed vertically in paraffin, sliced, dewaxed, and hydrated. Sections were stained with H&E for histopathological observation.

### 4.7. Biochemical Indicator Analysis

A quantity of 0.1 g skin tissue was ground into homogenate using phosphate buffer, and the supernatant was measured for ROS (Solarbio, CA1410), MDA (Solarbio, BC0025) and Hyp (Solarbio, BC0250) content according to the kit instructions.

### 4.8. RNA Extraction and qRT-PCR

After grinding the skin tissue (0.1 g) into powder, total RNA was extracted using TransZol reagent (TransGen Biotech, Beijing, China). RNA was processed using a reverse transcription kit (TransGen Biotech, Beijing, China) to obtain cDNA. According to the instruction of TransStart^®^ Green qPCR SuperMix, cDNA in a 20 µL reaction mixture was amplified using LightCycler^®^ 96 SW 1.1. The sequences of primers are listed in Table S1. The relative expression of genes was calculated using the 2^−ΔΔCT^ method [57].

### 4.9. Statistical Analysis

IBM SPSS version 22.0 (Chicago, IL, USA) and GraphPad Prism 9.0 (Inc., La Jolla, CA, USA) were used for data analysis. One-way analysis of variance (ANOVA) with Tukey’s test were used to compare the experimental groups. * *p* < 0.05, ^#^ *p* < 0.05 was considered statistically significant. Data results are expressed as mean ± standard deviation.

## 5. Conclusions

In this study, we extracted MAAs from the Antarctic diatom *Phaeodactylum tricornutum* ICE-H, which were composed of 4-DG, shinorine, and porphyra-334. These MAA compounds exhibited high free radical scavenging activity, alleviated the acute skin damage of mice caused by UVB, and decreased the degradation of collagen in the damaged skin. The protective mechanism of MAAs may be the reduction in UVB-induced ROS generation and oxidative stress, thereby inhibiting the activation of NF-κB and MAPK signals. Therefore, the MAA compounds from *Phaeodactylum tricornutum* ICE-H have the potential to be used as a photodamage treatment and cosmetic ingredient.

## Data Availability

Not applicable.

## Supplementary Materials

The following supporting information can be downloaded at: https://www.mdpi.com/article/10.3390/ijms242015055/s1.
