# Supplementary material for: Protective Effect of Mycosporine-like Amino Acids Isolated from an Antarctic Diatom on UVB-Induced Skin Damage
Source: Int J Mol Sci. 2023 Oct 10;24(20):15055. doi: 10.3390/ijms242015055 (PMC10606268; doi:10.3390/ijms242015055)
Supplement: Supplementary file 1 [file ijms-24-15055-s001.zip › Table S1.pdf]

Table S1. The primer sequences of quantitative real-time polymerase chain reaction.

Gene	Forward primer	Reverse primer
β-actin	CTACCTCATGAAGATCCTGACC	CCAGACACGGACAAGGCACAC
NF-κB*	CGGACGAGACGGATAGGCAGAG	CCAGACACGGACAAGGCACAC
COX-2*	ATTCCAAACCAGCAGACTCATA	CTTGAGTTTGAAGTGGTAACCG
TNF-α*	CACCACGCTCTTCTGTCTACTGAAC	AGATGATCTGAGTGTGAGGGTCTGG
IL-1β*	CTTCTTGGGACTGATGCTGGTGAC	TCTGTTGGGAGTGGTATCCTCTGTG
MMP-1*	ACAGTTGACAGGCTCCGAGAAATG	CCACATCAGGCACTCCACATCTTG
MMP-9	CGCCACCACAGCCAACTATGAC	CTGCTTGCCCAGGAAGACGAAG

* NF-κB, Nuclear factor-κB; COX-2, cyclooxygenase-2; TNF-α, Tumor necrosis factor-α; IL-1, Inter-leukin 1; MMP-1, Matrix metalloproteinases-1.
